# Identification of medicinal plant parts using depth-wise separable convolutional neural network

**DOI:** 10.1371/journal.pone.0322936

**Published:** 2025-05-07

**Authors:** Misganaw Aguate Widneh, Amlakie Aschale Alemu

**Affiliations:** Electrical and Computer Engineering, Gafat Institute of Technology, Debre Tabor University, Debre Tabor, Ethiopia; Regional Labor Institute, Directorate General Factory Advice Service and Labour Institutes (DGFASLI), Ministry of Labour and Employment, Government of India, INDIA

## Abstract

Identifying relevant plant parts is one of the most significant tasks in the pharmaceutical industry. Correct identification minimizes the risk of mis-identification, which might have unfavorable effects, and it ensures that plants are used medicinally. Traditional methods for plant part identification are often time-consuming and require specific expertise. This study proposed a Depth-wise Separable Convolutional Neural Network (DWS-CNN) to enhance the accuracy of medicinal plant part identification. Furthermore, we incorporated the tuned pre-trained model**s** such as VGG16, Res Net-50, and Inception V3 which are designed by Standard convolutional neural network (S-CNN) for comparative purposes. We trained variants of the Standard convolutional neural network (S-CNN) model with high-resolution images of medicinal plant leaves which contains 15,100 leaf images. The study used supervised learning by which leaf images are used as an identity for the other parts of the plants. We used transfer learning to tune training and model parameters. Experimental results showed that our DWS-CNN model achieved better performance compared to **S-**CNN models, with an accuracy of 99.84% for training data, 99.44% for F1-score and 99.44% for testing data, which improves in both accuracy and training speed. The presence of depth-wise separable convolution and batch normalization at the fully connected layer of the model made the model achieved a good classification performance.

## 1. Introduction

According to the World Health Organization statistics and other Literatures, about 80% of the population in some Asian and African countries depends on medicinal plants and herbs for their primary health care [1,2]. To produce the medicine from medicinal plant, physician should pass four steps. First, they should identify medicinal plants. At the second step, they should identify particular plant parts. Third, the active ingredients need to be extracted and finally the medicine will be produced. The physician should finish the first two steps before moving on to the final two steps. Time and effort are expended in the first two steps. Hence, physician's time and effort will be reducing in half by digitizing the first two phases. Identifying medicinal plants was not the aim of this study, rather we have collected known medicinal plants to identify their specific parts using DWS-CNN. Because only knowing medicinal plant needs further time and effort to identify particular part of the plant.

Deep learning can be used to, process natural Language, recognize speech and audio, bioinformatics, and identify medical image [3,4]. A deep learning model was built by [3] to identify medicinal plants. The deep learning model was also used to identify herbal using their flower images by extracting color, shape, and different texture features from flower images [5]. For example, AyurLeaf was a deep learning model [6] to classify medicinal plants using leaf features such as shape, size, color, texture, etc. However, it is not easy to get a distinct attribute of leaves using only the shape, color, and texture either separately or by combination. So, extracting vain and contour features using DWS-CNN is the better method to classify medicinal plant parts. To identify medicinal plant species uniquely, the vein and contour of the leaf are good features [7].

The ability of plants to cure disease using its parts and the need for improvements of indigenous knowledge to modern medical science are the main points for the excitement to proceed with this study. Hence, we proposed a fine-tuned DWS-CNN model using the transfer learning [8–10] technique for multi-class classification. VGG16, ResNet-50, and Inception V3 also experimented with fine-tuned models with the same data. Batch size, epoch, learning rate, and optimizer are the common training hyper-parameters tuned to get good identification results. Using batch size, we determined the number of training samples used in one iteration (epoch). Epoch helps us to determine the number of passes to describe how many trials a machine can learn from input data. We also used the learning rate to determine the step size in each iteration. We minimize training losses using optimizers by which the weight parameters are updated during back-propagation.

This paper is organized as follows: A literature review on medicinal plant identification, Depth-wise Separable convolutional neural network (DWS-CNN), and transfer learning are discussed in **section two**. A detailed description of the proposed methods is incorporated in **section three**. The study findings are discussed in **section four**. The study conclusion is **section five** of this paper.

## 2. Related works

Various image processing, machine learning (ML), and deep learning (DL) algorithms have been extensively used for the identifications of plant parts. In this section, a review of related works on identifications of plant parts and other related plant leaves are discussed.

Texture and color features were extracted from plant leaf images to develop a medicinal plant data set [11]. To extract medicinal plant features, the researchers used a classical machine learning technique such as a Decision Tree classifier, K-nearest neighbor's classifier, Weighted K-nearest neighbors classifier, Random Forest classifier. Subsequently [12] presented a fully automated method for the recognition of medicinal plants using computer vision and machine learning techniques. Leaves from 24 different medicinal plant species were collected. A large number of features were extracted from each leaf such as its length, width, perimeter, area, number of vertices, color, perimeter, and area of hull. With an accuracy of 90.1%, the random forest classifier performed better than other machine learning approaches. In the study [11], they discussed about the formation of the feature set which was an important step in recognizing any medicinal plant species. The vision-based approach was being employed to create an automated system that identifies the plants [13] also presented the medicinal plant leaves recognition approach using a neural network. The purpose of this work was to examine neural networks and their emerging applications in the field of engineering. Another notable work was proposed by [14] to extract medicinal plant information using a knowledge transfer where the raw plant leaf image was represented in deep features. These deep features are experimentally proven to outperform the state of the art in plant species recognition. A real time identification of medicinal plant based on the input leaf sample was proposed by [15]. The authors used ExG-ExR to obtain more vegetative information from the images. The plant species are classified by the color and texture features on each extracted leaf using a Logistic Regression classifier with an accuracy of 93.3%.

In recent study, medicinal plant leaf was used by [16] to assessed the performance of seven advanced deep learning algorithms and to suggest the best model from a comparative study of the models. They meticulously trained VGG16, VGG19, DenseNet201, ResNet50V2, Xception, InceptionResNetV2, and InceptionV3 deep neural network models. This training utilized a dataset comprising 5878 images encompassing 30 medicinal species distributed among 20 families. Furthermore, medicinal plant was identified by [17] using deep learning model and advanced computer vision. This study compares the Convolutional Neural Networks (CNN) variants with Mobile Net, ResNet50, Inception v3, Xception, and DenseNet121 for Indian-origin medicinal plant species detection. They adopted the Inception v3 model and the stochastic gradient descent technique during the training process for optimizing and achieving better results. The results show that the Inception v3 model achieved 95% accuracy. Authors in [18] also presented a deep learning-based methodology for the identification of medicinal plants. In the model development process, they preferred 7 pre-learning deep learning algorithms and an image data set created from plant leaves in ten categories to achieved test accuracy of 67.92% using Dense Net 121 model. According to [19], a robust, accurate, and practical system was presented to identify medicinal plants. The proposed system utilized a cascaded architecture to extract features using a pre-trained ResNet50 model, which was optimized using Particle Swarm Optimization (PSO) to classify the plants using a Support Vector Machine (SVM).

In [20] the depth-wise separable convolution model performed well in three benchmark hyper-spectral images (HIS) datasets such as Indian Pines, Pavia University, and Kennedy Space Center to classify hyper-spectral images. The author constructed a residual unit with fewer training parameters by combining the residual connection with the depth-wise separable convolution. A Depth-wise Separable Dense Convolutional Network with a Convolution Block Attention Module (AM-SdenseNet) also proposed by [21] to increase the speed and accuracy of COVID-19 diagnosis and achieved a model performance of 99.14. Furthermore, a depth-wise separable convolution (DSC) was proposed by [22] to replace the standard convolution to reduce the parameters and computational costs. Experimental findings show that the DSC algorithm, based on the OASIS magnetic resonance imaging dataset, was very successful in AD (Alzheimer’s disease) detection [23]. Replaced standard conventional encoder layers with depth-wise separable convolutions and transposed convolutional decoders with up-sampling plus depth-wise separable convolution. Their experimental results demonstrate substantial improvement over existing compact models in terms of state-of-the-art performance, while significantly reducing the number of parameters compared to larger models.

Depth wise separable convolution also used by [24] to detect micro-calcification clusters from mammograms and classification into malignant and benign classes. The authors designed a fully Connected Depth-wise Separable Convolutional Neural Network. The proposed method was evaluated on 3568 DDSM and 2885 PINUM mammogram images with automatic feature extraction, obtaining a score of 0.97 with a 2.35 and 0.99 true-positive ratio with 2.45 false positives per image, respectively. In [25], a Depth-wise Separable Convolution Attention Module (DSCAM) was used to overcome the limitations of existing garbage image classification methods. In DSCAM, the inherent relationships of channels and spatial positions in garbage image features are captured by two attention modules with depth-wise separable convolutions, so that their method can only focus on important information and ignore the interference. Authors of [26] proposed point-wise separable (PWS) and depth-wise separable convolutions, which are more efficient than standard convolutions. The proposed model outperforms all of the existing backbones for object detection and classification in three publicly accessible datasets: PASCAL VOC 2007, PASCAL VOC 2012, and MS-COCO. Their extensive experiments show that the proposed model outperforms state-of-the-art detectors, with mAP improvements of 2.4% and 2.5% on VOC 2007, 3.0% and 2.6% on VOC 2012, and 2.5% and 3.6% on MS-COCO in the small and large sizes of the images, respectively.

Several studies were presented for the identification of medicinal plants using Standard convolutional neural network (S-CNN) models such as [3,5,6,10,16], etc. However, those studies are not engaged in employing a DWS-CNN that can reduce the total trainable parameters and help the model achieve better accuracy by reducing over-fitting. They also did not reduce the computational load that occurred at the fully connected layer. Accordingly, we investigated and compared the performance of the DWS-CNN and S-CNN-based models towards the identification of the specific parts of medicinal plants. It is possible to extract leaf features directly from the raw representations of input data using depth-wise separable Convolutional Neural Networks (DWS-CNN). Many researchers have tried to develop a robust and efficient plant recognition system by exploiting pattern recognition and image processing techniques based on plant leaves, flowers, bark, and fruits. However, leaves play a vital role over other parts of a plant because they contain rich information as well as are more reliable [27–29]. So, this study particularly used the back side image of leaves of medicinal plants to accurately extract unique features.

Extensive studies focused on standard convolutional neural networks (S-CNN) without exploring the advantages of depth-wise separable convolutional neural networks (DWS-CNNs) while identifying medicinal plant using deep learning. Also, there are no researches aimed to address medicinal plant parts identification using DWS-CNN. The absence of batch normalization, which could reduce over-fitting and improve model generalization at the dense layer are also another key gap observed in the previous studies. So, this study investigated the effectiveness of DWS-CNN in identifying medicinal plant parts by comparing its performance with standard S-CNN. Additionally, batch normalization at the dense layer was incorporated to enhance model stability and accuracy. Hence, our study aims to contribute to optimizing CNN architectures in identification of medicinal plant parts. Some of the contributions are described as follows:

- The experiments are made on the proposed model and variants of the S-CNN model such as VGG16, Res Net-50, and Inception V3. Comprehensive comparisons are made among those models to evaluate the identification performances and the time taken to learn from the input data. Based on the experiment, all variants of S-CNN have lower performance and slower training speeds than the proposed model. Hence, we found the proposed model maintain a prominent solution for the identified problem.
- A combined operation of depth-wise and point-wise convolutions is used that reduces the total trainable parameters computed by scalar multiplication of kernels during feature extraction.
- In addition to the feature learning layer, a batch normalization layers are added at the dense layer making the proposed model learn faster and achieved higher testing accuracy by reducing complex mathematical operations that are the cause of over-fitting and computational load.
- Generally, our experiment discovered the advantages gained from the combined work of DWS-CNN and transfer learning that as faster training and higher medicinal plant part identification performance.
- At last, the proposed model is integrated with a mobile application that can identify medicinal plant parts from real-time photos as well as from gallery photos.

## 3. Methods and materials

An extensive study has been conducted using machine learning algorithms, including ANN, K-NN, SVM, Random Forest, Decision Tree, and standard convolutional neural network (S-CNN), to identify medicinal plants. In classical machine learning, a handcrafted feature extraction strategy resulted in a small number of feature maps in the training data. The model performed worse when a big training parameter was computed using S-CNN. To build a DWS-CNN model and compare it with S-CNN models like VGG16, Res Net-50, and Inception V3. The proposed approach automatically extracts couter, back vein, and other relevant structures from medicinal plant leaves. Once distinctive features are automatically extracted, we trained DWS-CNN and S-CNN to categorize medicinal plant parts based on multi-class categories. To improve feature uniqueness and get an accurate representation of the vein structure feature, the study leveraged the backside of the leaves.

### 3.1. Study procedure

**Fig 1:**
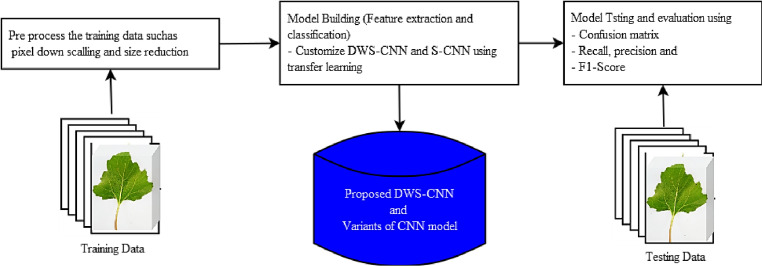
High-level study procedures.

To come up with a better experimental result, we followed scientific approaches such as data collection and annotation, feature extraction and training, and finally model testing. Data collection and data annotation (labeling) were performed before by [26]. Fig 1 shows the overall work procedure of the study. The image processing phase contains image size reduction and data normalization. At the model-building phase, a pre-trained model (DWS-CNN model) is adopted using a transfer learning technique which includes feature extraction and training.

### 3.2. Dataset and preprocessing

The data is collected from a local place of Ethiopia using a high-resolution camera (SAMSUNG A30S with 25MP and TECHNO SPARK K7 with 13MP) [28]. Different light intensities and orientations of the leaf are also considered. This helps to avoid time consumption to augment our data.

**Fig 2:**
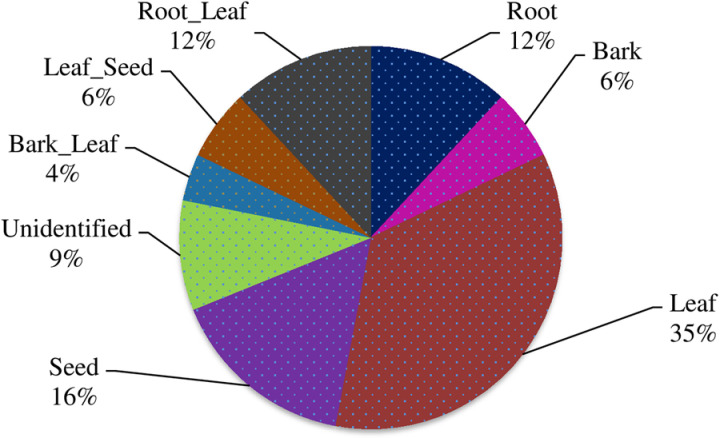
Data distribution in each target class.

Fig 2 shows the distribution of data samples in each category. This illustrates we have imbalanced data in each class. In general, we used 15,100 medicinal plant leaf photos.

**Fig 3:**
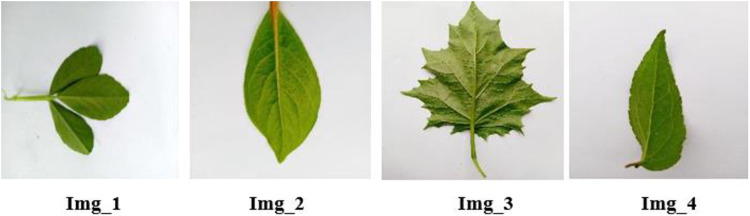
Sample leaf images.

According to [28] and [30], the data annotation (labeling) is supported by five experts who have 5, 10, 13, 28, and 30 years of experience and currently working on traditional medicine preparation. Fig 3 showed that, all parts of the plants are tagged (mapped) on the leaves image. That means our study was based on a supervised learning approach. Img_1, img_2…. img_n represents the file names of the leaf images. In the table column, the underscore sign was used to separate two labels. For instance, **leaf*_seed*** means the medicinal plant is used for medicine using its ***leaf*** and ***seed*** parts as indicated in Table 1. The model used the leaf image features as the identity of the corresponding plant parts or labels. That means the target labels are labeled (tagged) with an identity image even though those labels are not part of the image.

**Table 1:** Samples of target label mapping.

File name	Tags	Seed	Root	Leaf	Seed	Un identified	Bark_leaf	Leaf_seed	Root_leaf
img_1	[’ seed’]	1	0	0	0	0	0	0	0
img_2	[’root]	0	1	0	0	0	0	0	0
img_3	[’leaf_seed’]	0	0	0	0	0	0	1	0
img_4	[bark_leaf]	0	0	0	0	0	1	0	0

Even though a Google collaborator provides us with 12 GB of free computational resources, the input image was resized to 128X128 to save computing resources and time. In this stage, the input image pixels are scaled down to reduce image pixels between 0 and 1. Because learning step can be slow down when processing large-sized images with a large integer value (0–255). Ultimately, preprocessed image feature values are stored in a single variable for preserving data loading time.

### 3.3. Feature extraction and classification

Feature extraction and classification are the main tasks of the CNN while it is on training. We built a fine-tuned DWS-CNN model using the transfer learning technique. The fine-tuned models are pre-trained using the image net. In this procedure, all convolutional bases (feature learning layer) are frozen except the last two layers of the convolutional base which are trained using the new weights. All layers of a convolutional base in the designed DWS-CNN are followed by batch normalization and ReLU activations.

The classification layer of a model contains a flattened, fully connected layer, and a Softmax activation layer. The categorical cross entropy was used as a loss function. Because, at the last layer, there was a Softmax classifier to select one category from many classes.


$$f(Xi)=\frac{e^{Xi}}{\sum_{j=1}^{k}e^{Xj}}\;$$ (1)

Where ‘**f**’ is Softmax, ‘**xi**’ is the input vector, ‘**exi**’ is the standard exponential function for the input vector, and ‘**exj**’ is the standard exponential function for the output vector and number of classes in multi-class classification.

At this layer, two fully connected layers and batch normalization layers are added for both DWS-CNN and variants S-CNN. The advantages gained from this batch normalization were the model learned fast and achieved desired testing accuracy. So, in addition to layer tuning (customization), the deep learning hyper-parameters are tuned to come up with prominent classification results.

### 3.4. Model performance evaluation

Accuracy, Precision, Recall, and F-Score [31] were used for evaluating the model. The equations are mentioned as follows respectively. However, in this study, there was an imbalanced dataset among categories. So, F1_Score was a better metric to measure the performance of models [27] due to its harmonic mean value of recall and precision.


$$Accuracy=\frac{TP+TN}{TP+TN+FPFN}$$ (2)

$$Precision=\frac{TP}{TP+FP}$$ (3)

$$Recall=\frac{TP}{TP+FN}$$ (4)

$$F1_{Score}=\frac{2*Precision*Recall}{Precision+Recall}$$ (5)

### 3.5. Proposed model architecture using transfer learning

**Fig 4:**
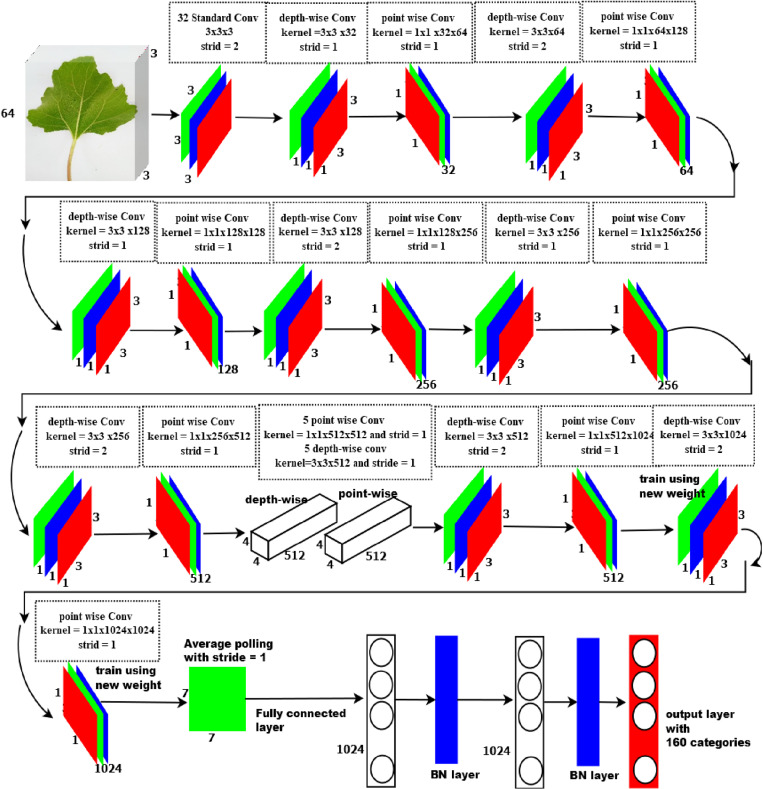
Architecture of proposed DWS-CNN (finetuned Mobile Net).

We did not build the model from scratch. Instead, a pre-trained model (Mobile Net) was customized using the transfer learning technique. Initially, this model was built for object detection purposes. Now we customized it by adding two dense layers and batch normalization layers at the fully connected layer. The advantages gained from this transfer learning were that the model learned using small training data and time, no need for a large number of training epochs. Fig 4 shows the architecture of the proposed model using depth-wise separable convolution (DWS-CNN). The depth-wise convolution used one filter for each color channel. To combine the outputs of the depth-wise convolution, the point-wise convolution then applied a 1 × 1 convolution. A standard convolution uses both filtrations and input combinations into a new set of outputs in one step. Standard convolution (S-CNN) computed huge parameters that made the model consume time to learn and overfit. So, leveraging the depth-wise separable convolution reduced the problem of S-CNN by splitting the convolution process into two layers for filtering and combining. This separation radically reduced the total computed parameters and model size. The original feature learning (extraction) layers of DWS-CNN are followed by batch normalization and ReLU activations except the classification layer. We trained the last two convolutional layers with new weights. However, there was a complex mathematical operation at a fully connected layer that was the cause of overfitting and computational load. Hence, we added two Batch normalization layers at the fully connected layer to reduce the defined problem.

#### 3.5.1. Operations of DWS-CNN.

**Fig 5:**
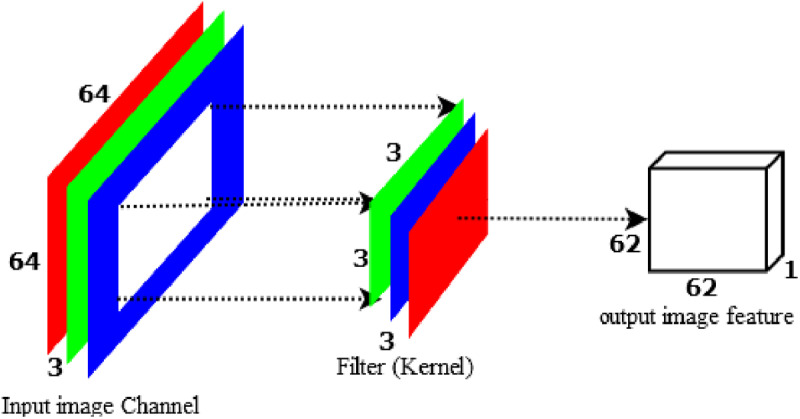
Standard convolution to produce one 8x8 output feature.

Before starting DWS-CNN, let us see how a standard convolution network (S-CNN) computes training parameter. As an illustration, let us give an input-colored image with a size of 64 by 64 to the S-CNN by applying a 3x3 convolution with a stride of 1 and no padding to the image. If we simply take the image's width: 64x64 with 3x3 kernel = 62x62. Every 9 pixels, the 3x3 kernel goes into scalar multiplication, providing a single integer each time. The 62x62 pixel image is obtained without padding (64–3)/1 + 1 = 62. The colored image, however, has three channels. Therefore, three channels are required for the convolutional kernel. Accordingly, the model computes 3x3x3=27 multiplications each time instead of 3x3=9. After passing a 3x3x3 kernel through a 62x62x3-sized image, the convolution layer produces a 62x62x1 image feature map as indicated in Fig 5.

**Fig 6:**
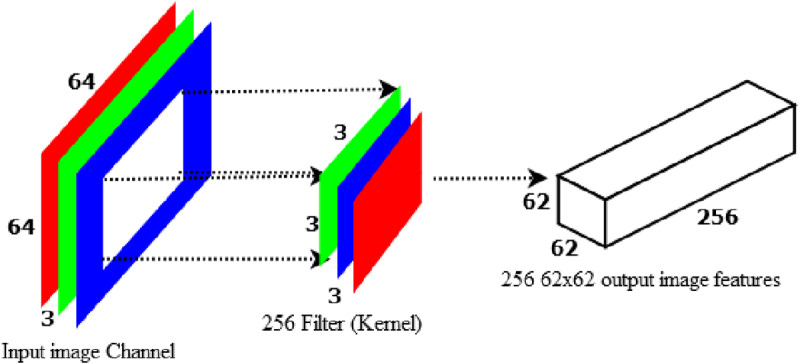
Standard convolution to produce 256 8x8 image features.

If we employ 256 filters with a size of 3x3x3 to obtain 256 62x62x1 image features, the model will calculate (3x3x3) * 256 * (62x62x1) = 26,569,728 parameters as indicated in Fig 6.

**Fig 7:**
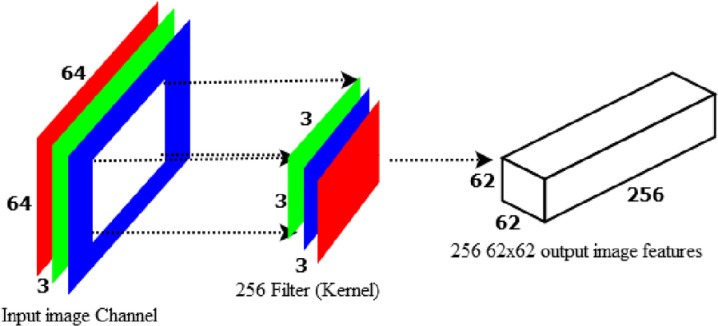
Depth-wise convolution to produce 3 channel 8x8 image feature.

Fig 7 indicated that, a 64x64x3 image has transformed to 62x62x256 image features using the standard convolution method. However, using depth-wise convolution, the 64x64x3 image can be converted to 62x62x3 image features.

**Fig 8:**
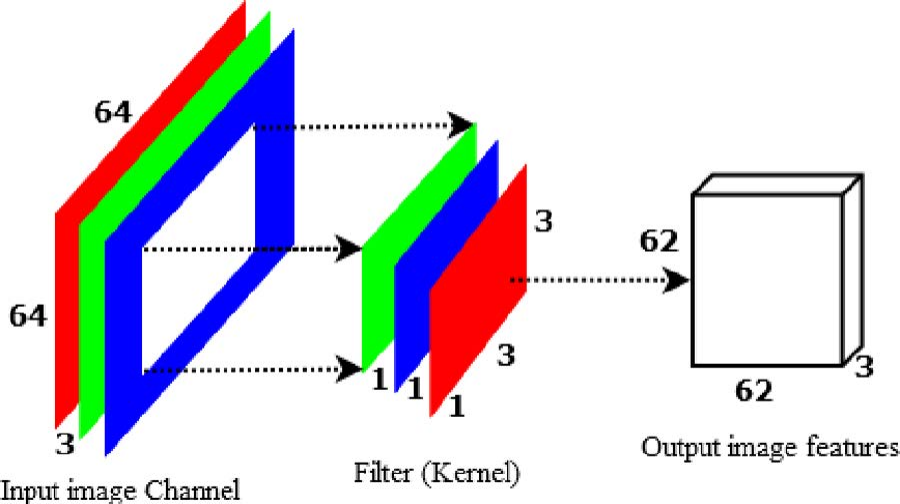
Point-wise convolution to produce one 8x8 image feature.

Every 3x3x1 kernel generates a 62x62x1 image by repeating through one channel of the image and obtaining the scalar products of each group of 9 pixels. A 62x62x3 image is made by layering these images together. To build a 62x62x1 image, DWS-CNN applies the 1x1x3 kernel across a 62x62x3 image as indicated in Fig 8. The point-wise convolution obtains its name due to the fact it applies a 1x1 kernel that iterates over every point. This kernel contains a depth of three channels in the input image.

**Fig 9:**
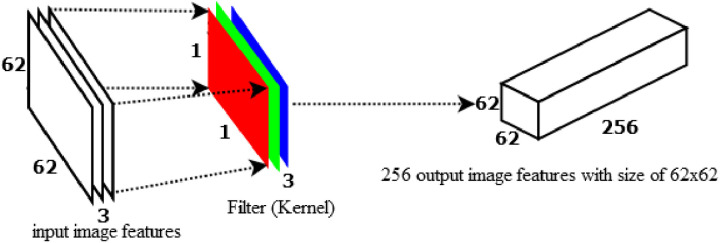
Point-wise convolution to produce 256 8x8 image features.

If we employ 256 filters with size of 3x3x3 to obtain 256 62x62x1 image features, the model will calculate (1x1x3) * 256 * (62x62x1) = 26,569,728 parameters. As indicated in Fig 9, DWS-CNN applied depth-wise convolution using 3 filters with a size of 3x3 to create 62x62x3 and produce 331,776 parameters. If we apply point-wise convolution using **256** filters with a size of 1x1x3 to create 256 62x62x1 image features, the model will calculate (3x3x3) * 256 * (62x62x1) = 3,145,728 parameters. Hence, the total parameter to be computed by Depth-wise separable convolution will be 331,776 + 3,145,728 = 3,477,504 parameters.

Based on the above explanation, the model can compute 26,569,728 and 3,477,504 total parameters for standard and depth-wise separable convolution respectively. Hence, this huge difference made the model designed by DWS-CNN lean fast and get higher testing accuracy.

## 4. Results and discussion

Table 2 indicates the parameters used in the study experiment that we have performed the experiments on a proposed model (DWS-CNN) and S-CNN models using 15,100 leaf images of medicinal plants. All the experimental results discussed in this section were obtained based on the following training parameters. After attempting many parameters by try and error, we have got these parameters that made the model learn better from the input data.

**Table 2:** The parameters used in the study experiment.

Parameters	Settled value
Optimizer	Adamax
Learning rate	1x10^-4^
Batch Size	32
No. of epochs	30

### 4.1. Experimental setup

All image data with the corresponding labels were imported to Google Drive and the data was further mounted to Google Collaborator. This study used Collaborator for experimental analysis such as feature extraction, training and testing. The data was split into 80% for the training set and 20% for the testing set. We used 3,020 images for testing and the remaining 12,080 data are for training from 15,100 leaf images of medicinal plants. Except for the last two layers, we frozen all feature learning layers of the proposed DWS-CNN and S-CNN (VGG16, ResNet-50, and Inception V3). We also added a new fully connected layer and batch normalization layers on the fine-tuned DWS-CNN. Training and model hyper-parameters are tuned to come up with a prominent solution. We used a batch size of 32 which determines the amount of data to be processed by the model at one time before updating the parameters. Employing a very large batch size needs higher memory capacity. Hence, we preferred the optimum size of batches. Leveraging very small and large values of learning rate made the training speed slower and faster without stable global minima respectively. So, we used the optimum learning rate value; which was 1x10^-4^. We ensured the reproducible result of the model by setting the random generator seed value of 42. At the last layer of the feature extraction part of CNN, average polling was used to reduce the dimension of the feature map to produce a smaller output feature. The training and model hyper-parameters and the type of convolution such as DWS-CNN and S-CNN are used for evaluation benchmarks of the proposed model. We used testing accuracy, confusion matrix and F1-score to measure the performance of the model.

**Fig 10:**
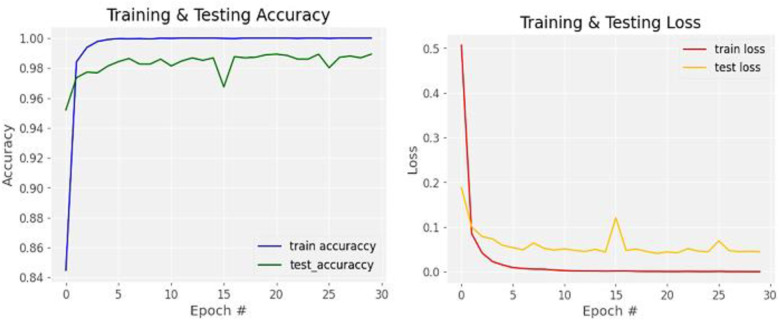
Accuracy and loss of a model for training and testing datasets.

This study used a dataset collected by [28] to train and test DWS-CNN (proposed model) and S-CNN. These models were adopted by tuning training and model hyper-parameters to achieve the goal. Fig 10 shows the best-case accuracy and loss of the proposed model. As shown in the figure, the training and testing accuracies start from higher values due to the application of transfer learning. That means the model tuned has been pre-trained by ImageNet that contains millions of plant leaf images which is one category among 1000 classes. Hence, before this study, the model already knows the common features of plant leaves. When transfer learning was used, the common features of plant leaves were not new for the fine-tuned pre-trained models. That is why the model starts its accuracy from higher values and learns fast as shown in Fig 10.

### 4.2. Model accuracies

The critical question during model building and training is how much it is effective when tested by the new (unseen) data. The testing accuracy can accurately determine the achievement of the models. Tables 3 and 4 shows the testing accuracies of the models trained using learning rates of 1x10^-4^ and 1x10^-3^ respectively. Both tables showed that the proposed model achieved a surpassed testing accuracy of 99.44% and 98.75% using the optimizer of Ada-max respectively.

**Table 3:** Testing accuracies of adopted models using learning rate 1*10^-4^.

Optimizers	Proposed Model	VGG16	Inception Net V3	Res Net- 50
Adam	99.27	99.07	90.53	80.36
Ada-max	99.44	99.01	88.37	84.40

**Table 4:** Testing accuracies of adopted models using learning rate 1*10^-3^.

Optimizers	Proposed Model	VGG16	Inception Net V3	ResNet-50
Adam	98.58	97.30	90.53	75.93
Ada-max	98.97	97.65	88.87	74.27

Generally, by looking through the result analysis, the proposed model was a good and fastest identifier. Because the model was designed by depth-wise separable convolution layers that make the model learn fast and reduce the problem of over-fitting. The depth-wise separable convolution makes the model reduce the total scalar multiplications produced by the convolution process.

### 4.3. Performances of the proposed model

**Fig 11:**
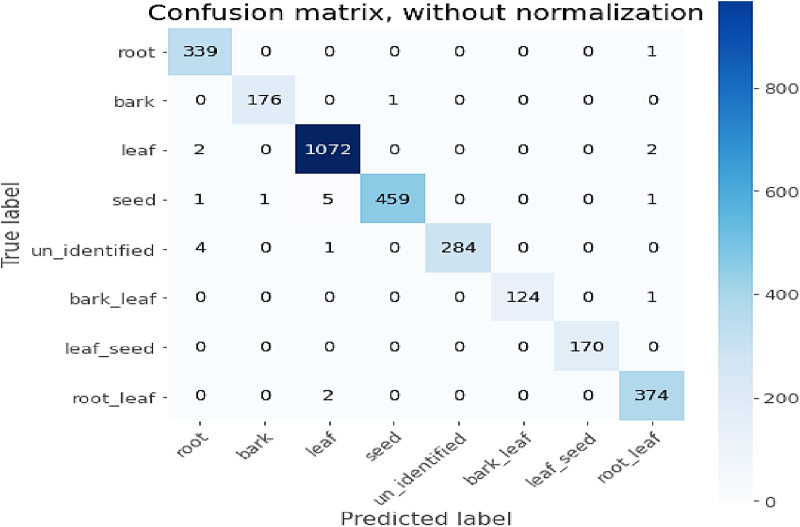
Confusion matrices of Proposed Model (Tuned DWS-CNN) for eight categories.

This study used a confusion matrix to evaluate the performance of a model using the test data by observing how many test samples of the data are classified correctly and how many are mis-classified in each class (category). Fig 11, Tables 5 and 6 are taken from the experimental result of the proposed model. We used only the classification report of the proposed model. As shown in Tables 3 and 4, the model outperformed the remaining three S-CNN models (VGG16, ResNet-50, and Inception V3).

**Table 5:** Correctly classified and misclassified classes performed by the proposed model (DWS-CNN).

Classes	Testing sample	Correctly classified	Miss classified
Root	340	339	1
Bark	177	176	1
Leaf	1076	1072	4
Seed	467	459	8
Un_identified	289	284	5
Bark_leaf	125	124	1
Leaf_seed	170	170	0
Root_leaf	376	374	2

**Table 6:** Classification report of the proposed model (Fine-tuned DWS-CNN) for eight categories.

	Precision	Recall	F1-score	Support
Root	98	100	99	340
Bark	99	99	99	177
Leaf	99	100	99	1076
Seed	100	98	99	467
Un_identified	100	98	99	289
Bark_leaf	100	99	100	125
Leaf_seed	100	100	100	170
Root_leaf	99	99	99	376
Accuracy			99	3020
Macro avg	99	99	99	3020
Weighted avg	99	99	99	3020

Fig 11 shows how much a model learned from each category. Based on this figure, Table 5 describes how many of the sample data are classified correctly (TP) and mis-classified (FP) in each of the Eight classes. Let us take the root of the plant as an illustration of the confusion matrix. It has a true positive value of 339 and a false-positive value of 1. That means the root sample has test data of 340. Among these data, 339 samples are categorized into the root correctly. Only 1 sample was misclassified as **root_leaf**. This showed the desired result of the classification task computed by the proposed model.

Table 6 shows the classification report performances of the proposed model for individual class. The study took the value of F1_score for the discussion; because this evaluation metric took advantage of both precision and recall by computing their harmonic mean. The F1_score result showed that the model attained desirable performances in both classes. Especially for two categories, the model achieved F1-Score values of 100%. It is also possible to make sure by referring the Table 5. Table 5 shows how the model performed with 0 and 1 mis-classification for leaf_seed and bark_leaf categories respectively. The identification results in the prediction section are also evidence of the performance of the proposed model.

**Fig 12:**
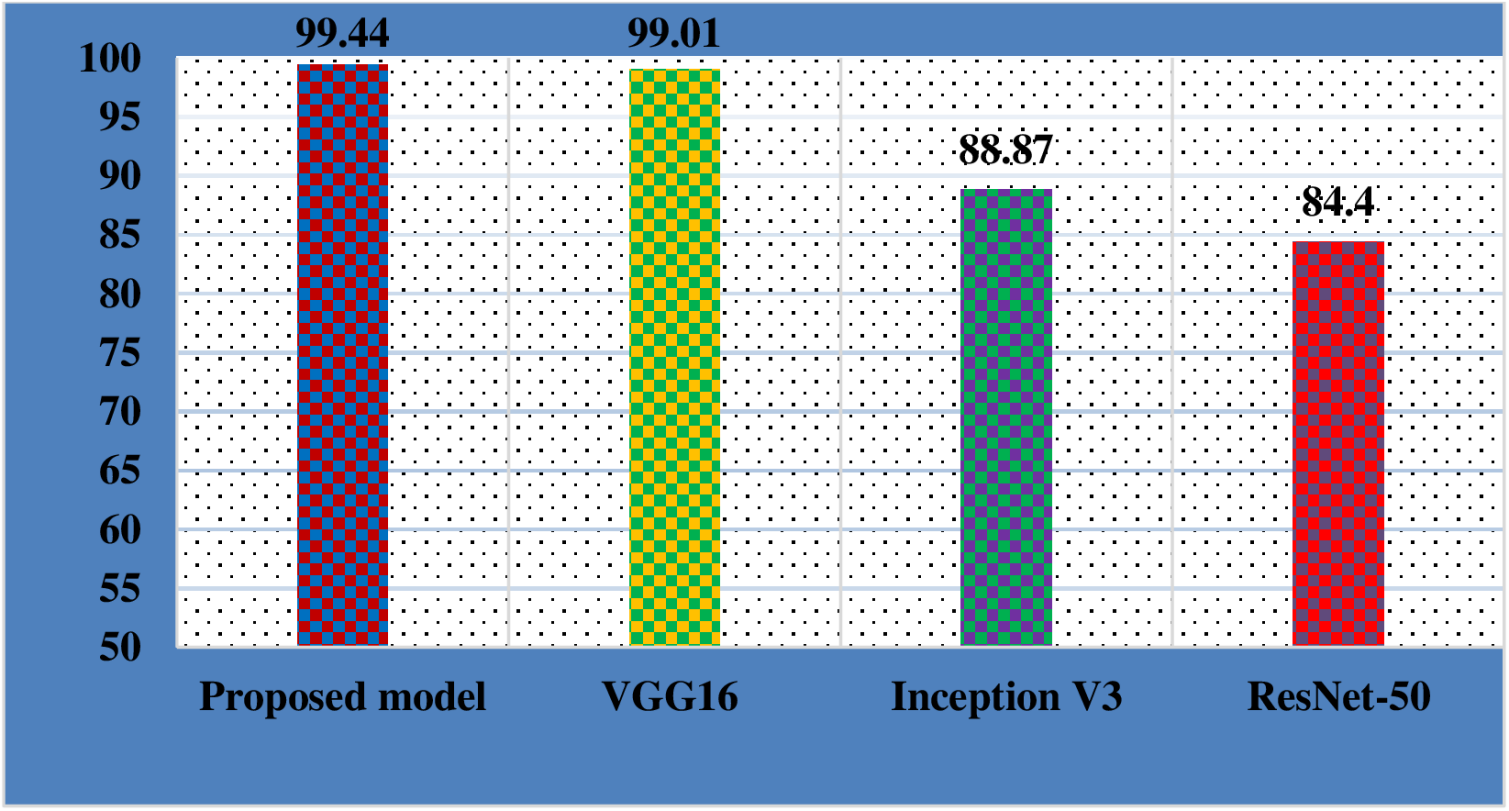
F1_score value of the models using learning rate  = 1*10-4 and batch size of 32.

To evaluate the effectiveness of the proposed DWS-CNN model and S-CNN models thoroughly, it was recommended to consider multiple evaluation metrics. These metrics provided us different insights for the model's performances and helped to made an overall measurement of the effectiveness of predictions. Table 6 shows the classification report of the model. This report narrates the performance of the proposed model for each class when measured by recall, precision, and F1-Score. But except for F1-Score, all metrics were not an effective evaluation metrics when there was an imbalanced dataset. Our dataset has imbalanced distribution of data in each category. Hence, F1_score was used as a better choice. The F1 Score was computed as a weighted average of the precision and recall values [32]. As indicated in Fig 12, the proposed model achieved a good classification performance over other S-CNN models. According to the experiment, DWS-CNN attained an F1-Score of 99.44%. It made the model well learned from the input data while the F1-Score of S-CNN (tuned VGG16) is 99.01% using the same input data and optimizer (Ada-max), ranking it as the second model.

a. Statistical testing accuray of the models: Table 7 illustrates different testing accuracy of the four models while tested using different optimizers and learning rates. We conducted statistical tests on testing accuracy across multiple runs of the models to confirm the significance of these accuracy differences. An ANOVA (Analysis of variation) test across all four models achieved a P-value of 2.332*10^-6^ which confirmed the differences are statistically significant and ensured that the performance improvement of our study was not random. Here P-value means the probability that the observed difference happened by chance. P-value < 0.05 means, there was a statistically significant difference between model accuracies.

**Table 7:** Testing accuracy of the models for each training.

Models	Optimizers			
	Adamax		Adam	
	Learning rate = 10^–3^	Learning rate = 10^–4^	Learning rate = 10^–3^	Learning rate = 10^–4^
VGG16	97.30	99.07	97.65	91.01
Inception Net	90.53	90.53	88.87	88.37
Res Net 50	75.93	80.36	74.27	84.40
Proposed model	98.58	99.28	98.97	99.44

Due to limited computational resources, we did not compute cross validation (K-fold method) for 15000 images. But we computed mean and standard deviation of accuracy and F1-score for each fine-tuned model. Using mean, we measured how well the models are performed on average while standard deviation determined the variabilities in models’ performance across multiple training. The lower value of standard deviation shown in Table 8 ensured the proposed model was consistently performed well while the higher values of Inception Net and Res Net 50 shows the performance fluctuates significantly between multiple training attempt.

**Table 8:** Mean and standard deviation of accuracy and F1-score of each model.

Models	Accuracy (%)	F1-Score (%)
Proposed model	99.065 ± 0.377	99.145 ± 0.290
VGG16	98.256 ± 0.915	98.250 ± 0.957
Inception Net	89.575 ± 1.121	89.750 ± 1.500
Res Net50	78.750 ± 4.582	78.500 ± 4.435

### 4.5. Time comparison

We utilized Google Collaboratory for experimental analysis. Hence, all models were run on this working platform. Table 9 shows the training time of the models from the dataset using iteration, optimizer, and Batch size with values of 30, Adamax, and 32 respectively. The result in the table showed that the proposed model learned faster over S-CNN-based models due to the computation of total parameters. As described in detail in the methods section, DWS-CNN computes a very small number of parameters compared to S-CNN. The training speed is directly determined by the total number of trainable parameters. We recorded the following time by making the last two feature learning layers of all models trained by new weights and adding two batch normalization layers at fully connected layers. This time variation occurred due to the type of convolution such as depth-wise separable convolution and standard convolution and depth of the CNN.

**Table 9:** Training time of proposed model and variants of CNN model.

Models	Training time (Minutes)
Proposed DWS-CNN Model	2.8
VGG16 (S-CNN model)	9.35
Inception Net V3 (S-CNN model)	5.28
ResNet-50 (S-CNN model)	3.30

Table 10, shows the comparative summary of the DWS-CNN and S-CNN-based model. Based on the table, the proposed model achieved higher testing accuracy over S-CNN works due to three main reasons. First, we used a larger amount of data. Second, we used DWS-CNN which can reduce the total trainable parameter computed by scalar multiplication during convolution. At last, we added batch normalization at a fully connected layer that reduced the computational load and over-fitting.

**Table 10:** Comparative analysis of previous work with the proposed method.

Related works	Medicinal plant data size	Variants of CNN	Testing accuracy
C. Amuthalingeswaran et al. [1]	8000	S-CNN	85
J. W. Tan et al. [2]	1290	S-CNN	95.54
M. R. Dileep and P. N. Pournami [4]	2400	S-CNN	96.76
F. Khalid and A. A. Romle [7]	2,093	S-CNN	97.08
S. Roopashree and J. Anitha [8]	2515	S-CNN	97.5
S. Prasad and P. P. Singh [33]	1500	S-CNN	98.2
B. Dey et al. [14]	5878	S-CNN	96.2
K. Kavitha et al. [15]	60	S-CNN	95
K. Kayaalp [16]	4503	S-CNN	98.69
Y. G. Naresh and H. S. Nagendraswamy [25]	1125	S-CNN	96.83
Misganaw Aguate et al. [26]	15100	S-CNN	94
Proposed model	15100	DWS-CNN	99.44

### 4.6. Integrating a model with a mobile application

**Fig 13:**
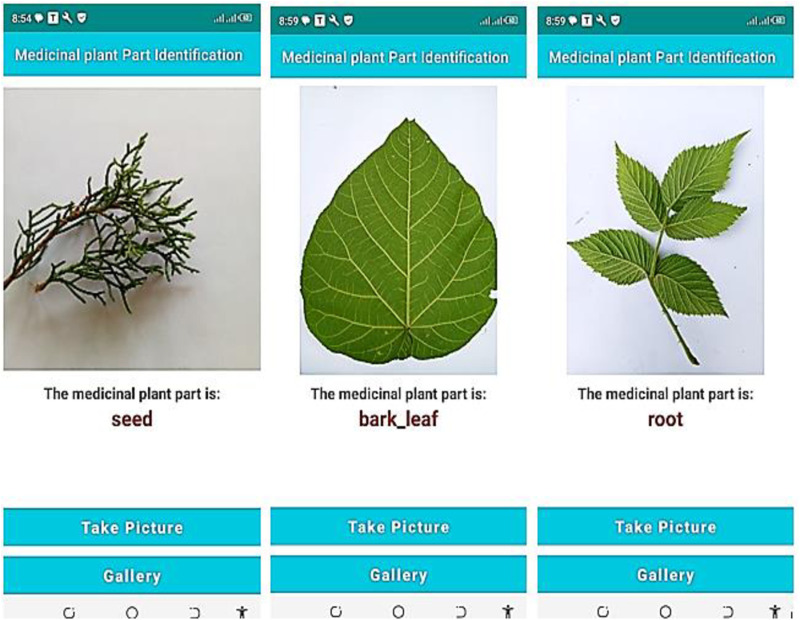
Predicted medicinal plant using the proposed model.

Integrating a deep learning model with a mobile application has a lot of potential for enhancing user experiences. So, we ensured the effectiveness of the proposed model by integrating the model with the mobile application. A question may be raised while observing Fig 13. “From the leaf image, how the model predicts another part of the medicinal plant?”. Here, the leaf image is used as the identity of the remaining parts. In supervised learning, a single image feature can be an identity for many true labels. Labeling (mapping) is assigning the target (true) values of the input image that we want from the classification result. Hence, even though other parts of the medicinal plant are not part of the training image, we tagged (mapped) many parts of the medicinal plant with leaf images as target labels. We simply developed this mobile Application to test whether the model that is converted to TFlite (TensorFlow Lite) model is work or not. To get the APK file, you can follow the following steps.

a. Browse https://project.ethioptec.com/
b. Click on Mobile Apps link found on the header.
c. Click on Download APK file on the Medicinal plant part App.

**Remember**: The model to be integrated with Mobile App should trained using huge dataset to be fully functional. But deploying a model trained using huge data needs higher performance computing devices to get the intended functionality. For functional model, we planned to collect huge data (thousands of plant species with millions of leaf images), build prominent model and deploy on Computer based application. For the model trained by Hug data, Mobile device will not applicable due to computational resource constraints.

## 5. Conclusion and future works

### 5.1. Conclusion

This study mainly emphasized on the identification of medicinal plant parts using the DWS-CNN model. As a result, a new fine-tuned model was built by making the last two layers of the convolutional base trained by new weight and adding two batch normalization layers at the dense layer of the pre-trained model. The study applied a transfer learning technique to DWS-CNN and S-CNN-based models. To build the model, 15,100 medicinal plant leaf images were used. We made performance and time comparisons among the proposed model and variants of CNN models. The experimental result showed that the proposed model achieved a high testing accuracy and F1-Score of 99.44% in identifying various parts of medicinal plants parts, such as seed, root, leaf, bark_leaf, leaf_seed, root_leaf, and un identified. This result indicates that, the proposed model was performed over variants of S-CNN. This model also needed 2.8 minutes to complete training; which is a smaller time compared to S-CNN models that consumed 9.35, 5.28, and 3.3 minutes for VGG16, Inception V3, and ResNet-50 respectively. This study also proved that the over-fitting of a model was affected by the total number of output parameters calculated using scalar multiplication of filters (kernel). In this study, the DWS-CNN model has a larger hidden layer than S-CNN (VGG16) and was trained by the same amount of data. But S-CNN (VGG16) model is over-fitted than DWS-CNN. Because DWS-CNN highly reduced the total trainable parameters using pointwise convolution while extracting and combining image features. The addition of batch normalization at a fully connected layer of the CNN model also highly determined the training speed and performance of the models by reducing computational load. Finally, we claim that the combined works of DWS-CNN and transfer learning is a better practice to get higher accuracy in shorter training speed for Medicinal plant parts identification.

### 5.2. Future works

This study only identifies the specific part of the plant that used for medicine. We did not incorporate the type of disease cured by the identified medicinal plant part. Because to incorporate the exact type of disease to be cured, there needs extracting active ingredients from the identified plant parts. Another constraint of this study was lack of data to deploy the model on computer application for real time application. It is better if the future work will incorporate three tasks. First, huge data (thousands of plant species with millions of leaf images) should be collected. Second, active ingredients should be extracted for all identified plant parts by cooperating pharmacy specialists. The last will be build R-CNN model and deploy on Computer based application. For the model trained by Hug data, Mobile device will not applicable due to computational resource constraints.

## References

[1] Nyirenda J, Chipuwa M. An ethnobotanical study of herbs and medicinal plants used in Western, Copperbelt, Central and Northern provinces of Zambia. Phytomedicine Plus. 2024;4(1):100514. doi: 10.1016/j.phyplu.2023.100514

[2] Ralte L, Sailo H, Singh YT. Ethnobotanical study of medicinal plants used by the indigenous community of the western region of Mizoram, India. J Ethnobiol Ethnomed. 2024;20(1):2. doi: 10.1186/s13002-023-00642-z 38172927 PMC10765666

[3] Amuthalingeswaran C, Sivakumar M, Renuga P, Alexpandi S, Elamathi J, Hari SS. Identification of medicinal plant’s and their usage by using deep learning. 2019 3rd International Conference on Trends in Electronics and Informatics (ICOEI). 2019. doi: 10.1109/icoei.2019.8862765

[4] Tan JW, Chang S-W, Abdul-Kareem S, Yap HJ, Yong K-T. Deep learning for plant species classification using leaf vein morphometric. IEEE/ACM Trans Comput Biol Bioinform. 2020;17(1):82–90. doi: 10.1109/TCBB.2018.2848653 29994129

[5] Bandara M, Ranathunga L. Texture dominant approach for identifying ayurveda herbal species using flowers. 2019 Moratuwa Engineering Research Conference (MERCon). 2019:117–22. doi: 10.1109/mercon.2019.8818944

[6] Dileep MR, Pournami PN. AyurLeaf: a deep learning approach for classification of medicinal plants. TENCON 2019 - 2019 IEEE Region 10 Conference (TENCON). 2019:321–5. doi: 10.1109/tencon.2019.8929394

[7] Lee SH, Chan CS, Mayo SJ, Remagnino P. How deep learning extracts and learns leaf features for plant classification. Pattern Recognit. 2017;71:1–13. doi: 10.1016/j.patcog.2017.05.015

[8] Zhuang F, Qi Z, Duan K, Xi D, Zhu Y, Zhu H, et al. A comprehensive survey on transfer learning. Proc IEEE. 2021;109(1):43–76. doi: 10.1109/jproc.2020.3004555

[9] Fatimah K, Amirul Azuani R. Herbal plant image classification using transfer learning and fine-tuning deep learning model. J Adv Res Appl Sci Eng Technol. 2023;35(1):16–25. doi: 10.37934/araset.34.3.1625

[10] Roopashree S, Anitha J. DeepHerb: a vision based system for medicinal plants using Xception features. IEEE Access. 2021;9:135927–41. doi: 10.1109/access.2021.3116207

[11] Pacifico LDS, Britto LFS, Oliveira EG, Ludermir TB. Automatic classification of medicinal plant species based on color and texture features. 2019 8th Brazilian Conference on Intelligent Systems (BRACIS). 2019:741–6. doi: 10.1109/bracis.2019.00133

[12] Kumar A, Kumar DB. Automatic recognition of medicinal plants using machine learning techniques. Gedrag Organ Rev. 2020;33(1):166–75. doi: 10.37896/gor33.01/012

[13] K N, Bhaskara RT. Analysis of the medicinal leaves by using image processing techniques and ann. Int J Adv Res Comput Sci. 2017;8(5):1801–5.

[14] Prasad S, Singh PP. Medicinal plant leaf information extraction using deep features. TENCON 2017 - 2017 IEEE Region 10 Conference. 2017:2722–6. doi: 10.1109/tencon.2017.8228324

[15] Sivaranjani C, Kalinathan L, Amutha R, Kathavarayan RS, Jegadish Kumar KJ. Real-Time Identification of medicinal plants using machine learning techniques. 2019 International Conference on Computational Intelligence in Data Science (ICCIDS). 2019:1–4. doi: 10.1109/iccids.2019.8862126

[16] Dey B, Ferdous J, Ahmed R, Hossain J. Assessing deep convolutional neural network models and their comparative performance for automated medicinal plant identification from leaf images. Heliyon. 2023;10(1):e23655. doi: 10.1016/j.heliyon.2023.e23655 38187334 PMC10767391

[17] Kavitha K, Sharma P, Gupta S, Lalitha RVS. Medicinal Plant Species Detection using Deep Learning. 2022 First International Conference on Electrical, Electronics, Information and Communication Technologies (ICEEICT). 2022:01–6. doi: 10.1109/iceeict53079.2022.9768649

[18] Kayaalp K. Classification of medicinal plant leaves for types and diseases with hybrid deep learning methods. Inf Technol Control. 2024;53(1):19–36. doi: 10.5755/j01.itc.53.1.34345

[19] Islam MdT, Rahman W, Hossain MdS, Roksana K, Azpíroz ID, Diaz RM, et al. Medicinal plant classification using particle swarm optimized cascaded network. IEEE Access. 2024;12:42465–78. doi: 10.1109/access.2024.3378262

[20] Dang L, Pang P, Lee J. Depth-wise separable convolution neural network with residual connection for hyper-spectral image classification. Remote Sensing. 2020;12(20):3408. doi: 10.3390/rs12203408

[21] Li Q, Ning J, Yuan J, Xiao L. A depth-wise separable dense convolutional network with convolution block attention module for COVID-19 diagnosis on CT scans. Comput Biol Med. 2021;137:104837. doi: 10.1016/j.compbiomed.2021.104837 34530335 PMC8425669

[22] Liu J, Li M, Luo Y, Yang S, Li W, Bi Y. Alzheimer’s disease detection using depth-wise separable convolutional neural networks. Comput Methods Programs Biomed. 2021;203:106032. doi: 10.1016/j.cmpb.2021.106032 33713959

[23] Wang T, Ray N. Compact depth-wise separable precise network for depth completion. IEEE Access. 2023;11:72679–88. doi: 10.1109/access.2023.3294247

[24] Rehman KU, Li J, Pei Y, Yasin A, Ali S, Mahmood T. Computer vision-based microcalcification detection in digital mammograms using fully connected depth-wise separable convolutional neural network. Sensors (Basel). 2021;21(14):4854. doi: 10.3390/s21144854 34300597 PMC8309805

[25] Liu F, Xu H, Qi M, Liu D, Wang J, Kong J. Depth-wise separable convolution attention module for garbage image classification. Sustainability. 2022;14(5):3099. doi: 10.3390/su14053099

[26] Junayed MS, Islam MB, Imani H, Aydin T. PDS-Net: a novel point and depth-wise separable convolution for real-time object detection. Int J Multimed Info Retr. 2022;11(2):171–88. doi: 10.1007/s13735-022-00229-6

[27] Naresh YG, Nagendraswamy HS. Classification of medicinal plants: an approach using modified LBP with symbolic representation. Neurocomputing. 2016;173:1789–97. doi: 10.1016/j.neucom.2015.08.090

[28] Aguate AAM, Tesfahun A. Medicinal plant part identification and classification using deep learning based on multi label categories. Ethiop J Sci Sustain Dev. 2021;8(2).

[29] Kaur S, Kaur P. Plant species identification based on plant leaf using computer vision and machine learning techniques. J Multimed Inf Syst. 2019;6(2):49–60. doi: 10.33851/jmis.2019.6.2.49

[30] Widneh MA, Alemu AA, Getie DD. Exploring Batch Normalization’s impact on dense layers of multi-class and classifiers. Int J Intell Syst. 2025;2025(1). doi: 10.1155/int/1466655

[31] Bekkar M, Djemaa HK, Alitouche TA. Evaluation measures for models assessment over imbalanced data sets. J Inf Eng Appl. 2013;3(10):27–38.

[32] Jeni LA, Cohn JF, De La Torre F. Facing Imbalanced Data Recommendations for the Use of Performance Metrics. Int Conf Affect Comput Intell Interact Workshops. 2013;2013:245–51. doi: 10.1109/ACII.2013.47 25574450 PMC4285355

[33] Venkataraman D, Mangayarkarasi N. Computer vision based feature extraction of leaves for identification of medicinal values of plants. 2016 IEEE International Conference on Computational Intelligence and Computing Research (ICCIC). 2016:1–5. doi: 10.1109/iccic.2016.7919637

